# Is there an effect of ghrelin/ghrelin analogs on cancer? A systematic review

**DOI:** 10.1530/ERC-16-0130

**Published:** 2016-09-01

**Authors:** Sakine Sever, Donna L White, José M Garcia

**Affiliations:** 1 Division of EndocrinologyDiabetes, and Metabolism, Baylor College of Medicine, Alkek Building for Biomedical Research, Houston, Texas, USA; 2 Section of Gastroenterology and HepatologyBaylor College of Medicine Medical Center, Houston, Texas, USA; 3 Clinical Epidemiology and Comparative Effectiveness ProgramSection of Health Services Research (IQuESt), Michael E. DeBakey Veterans Affairs Medical Center, HSR&D Center of Innovation (152), Houston, Texas, USA; 4 Texas Medical Center Digestive Disease CenterBaylor College of Medicine, Houston, Texas, USA; 5 Dan L. Duncan Comprehensive Cancer CenterBaylor College of Medicine, Houston, Texas, USA; 6 Center for Translational Research on Inflammatory Diseases (CTRID)Michael E. DeBakey Veterans Affairs Medical Center, Houston, Texas, USA; 7 Department of Molecular and Cellular BiologyBaylor College of Medicine, Houston, Texas, USA; 8 Huffington Center on AgingBaylor College of Medicine, Houston, Texas, USA; 9 Geriatrics Research Education and Clinical CenterVeterans Affairs Puget Sound Health Care System and University of Washington, Seattle, Washington, USA

**Keywords:** ghrelin, cancer, tumor growth, metastasis, *in vivo*, cachexia

## Abstract

Ghrelin is a hormone with multiple physiologic functions, including promotion of growth hormone release, stimulation of appetite and regulation of energy homeostasis. Treatment with ghrelin/ghrelin-receptor agonists is a prospective therapy for disease-related cachexia and malnutrition. *In vitro* studies have shown high expression of ghrelin in cancer tissue, although its role including its impact in cancer risk and progression has not been established. We performed a systematic literature review to identify peer-reviewed human or animal *in vivo* original research studies of ghrelin, ghrelin-receptor agonists, or ghrelin genetic variants and the risk, presence, or growth of cancer using structured searches in PubMed database as well as secondary searches of article reference lists, additional reviews and meta-analyses. Overall, 45 (73.8%) of the 61 studies reviewed, including all 11 involving exogenous ghrelin/ghrelin-receptor agonist treatment, reported either a null (no statistically significant difference) or inverse association of ghrelin/ghrelin-receptor agonists or ghrelin genetic variants with cancer risk, presence or growth; 10 (16.7%) studies reported positive associations; and 6 (10.0%) reported both negative or null and positive associations. Differences in serum ghrelin levels in cancer cases vs controls (typically lower) were reported for some but not all cancers. The majority of *in vivo* studies showed a null or inverse association of ghrelin with risk and progression of most cancers, suggesting that ghrelin/ghrelin-receptor agonist treatment may have a favorable safety profile to use for cancer cachexia. Additional large-scale prospective clinical trials as well as basic bioscientific research are warranted to further evaluate the safety and benefits of ghrelin treatment in patients with cancer.

## Introduction

Ghrelin is a 28-amino acid peptide with an *n*-octanoyl ester at its third serine residue, which is the endogenous ligand for the ghrelin receptor (formerly known as the growth hormone (GH) secretagogue receptor) and a hormone with multiple biologic functions (Kojima *et al*. 1999, Korbonits *et al*. 2004, Delporte 2013, Chen & Enriori 2015). Circulating ghrelin in humans consists of acylated (acyl) ghrelin and unacylated (des-acyl) ghrelin, which vary in their proportions over time, due in part to the rapid conversion of acyl to des-acyl ghrelin that appears to occur through circulating esterases (Tong *et al*. 2013, Delhanty *et al*. 2015). When acyl ghrelin was stabilized by esterase inhibition, the acyl to des-acyl ghrelin ratio was shown to range from 1:2 to 1:8 (Delhanty *et al*. 2015). Acyl ghrelin binds to the ghrelin receptor with 1000 times greater potency than des-acyl ghrelin, and is considered the only form capable of clinically relevant ghrelin-receptor activation; the term ghrelin (endogenous or exogenous) thus generally refers to the acyl or ‘active’ form (Bednarek *et al*. 2000, Matsumoto *et al*. 2001, Gauna *et al*. 2007). Nonetheless, des-acyl ghrelin appears to have multiple physiologic actions, including modulation (agonism or antagonism) of several of ghrelin’s actions (Delhanty *et al*. 2010, Chen & Enriori 2015) that do not require the presence of the ghrelin receptor. The existence of receptors specific to des-acyl ghrelin, as well as additional ghrelin receptors, has been proposed, but not yet demonstrated (Callaghan & Furness 2014).

Discovered in 1999, ghrelin was initially observed to stimulate pituitary release of GH in a dose-dependent manner (Kojima *et al*. 1999, Takaya *et al*. 2000), and later found to play an important role in the hypothalamic regulation of energy homeostasis by stimulating appetite and feeding through central and peripheral pathways, and via the vagus nerve (Nakazato *et al*. 2001, Williams & Cummings 2005, Delporte 2013). While 70% of circulating ghrelin is produced in the stomach, it is also expressed in diverse tissues, including the lungs, heart, intestines, pancreas, kidneys, gonads, pituitary and hypothalamus (Jeon *et al*. 2004, Delporte 2013). Circulating ghrelin levels increase under conditions of fasting or low body mass index (BMI) such as disease-related cachexia, anorexia nervosa and other states of malnutrition. Conversely, ghrelin levels decrease in response to rising BMI and obesity, and increased levels of glucose, insulin, lipids, leptin, GH, somatostatin, peptide YY, urocortin-1 and gastrin (Tschöp *et al*. 2001, Shiiya *et al*. 2002, Murdolo *et al*. 2003, Korbonits *et al*. 2004, Soriano-Guillén *et al*. 2004, Garcia *et al*. 2006, Ingelsson *et al*. 2008, Rau *et al*. 2013). Ghrelin levels decline with age, and are higher in women than in men (Korbonits *et al*. 2004, Ingelsson *et al*. 2008).

**Figure 1:**
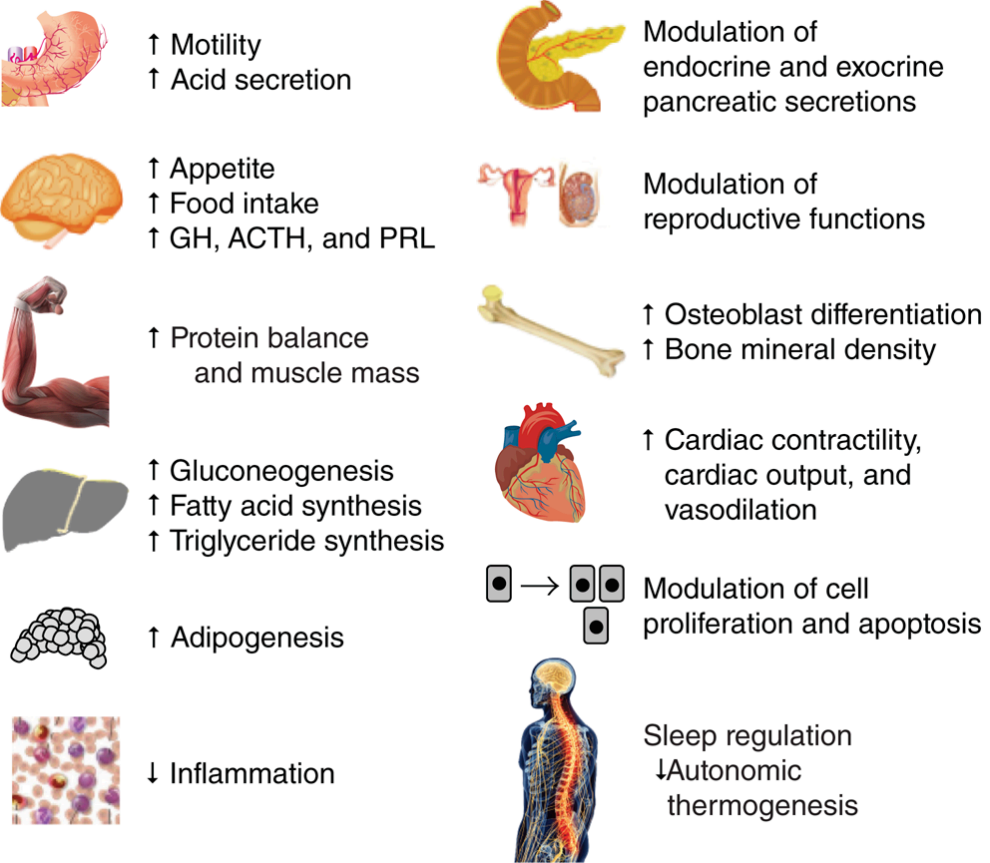
Physiologic effects of ghrelin. ACTH, adrenocorticotropic hormone; GH, growth hormone; PRL, prolactin. Adapted, under the terms of the Creative Commons Attribution License, from Delporte *et al*. 2013; additional data from Korbonits *et al*. 2004.

Ghrelin also modulates blood glucose levels and glucose disposal in skeletal muscle and adipose tissue in conjunction with GH and insulin-like growth factor 1 (IGF1), and regulates peripheral lipid metabolism and anabolic processes such as lipid storage via mainly GH-independent mechanisms (Nass *et al*. 2010, Varela *et al*. 2011). Collectively, these actions and characteristics suggest a prominent physiologic role for ghrelin as a regulator of energy balance and homeostasis (Korbonits *et al*. 2004, Williams & Cummings 2005, Varela *et al*. 2011, Chen & Enriori 2015). In addition, ghrelin appears to contribute through both GH-dependent and GH-independent pathways to regulation of the cardiovascular and reproductive systems, gastrointestinal function, pancreatic function, adipogenesis, angiogenesis, bone formation, anti-inflammatory and immune functions, muscle function and cell proliferation (Tschöp *et al*. 2000, 2001, Korbonits *et al*. 2004, Li *et al*. 2007, Bataar *et al*. 2011, Delporte 2013, Porporato *et al*. 2013, Chen & Enriori 2015) (Fig. 1). Some consequences of ghrelin dysregulation may be demonstrated in Prader–Willi syndrome, a neurogenetic disorder that is characterized by poor feeding and weight gain in early infancy followed by hyperphagia, impaired satiety, severe obesity, and multiple dysmorphic and psychocognitive developmental problems in childhood and adulthood. This disorder is associated with hyperghrelinemia and increased acyl to des-acyl ghrelin ratio (Feigerlová *et al*. 2008, Kuppens *et al*. 2015). Based on its actions in maintaining energy homeostasis and promoting adipogenesis and muscle function, ghrelin/ghrelin-receptor agonist therapy is considered to have promising potential for restoring energy homeostasis in conditions such as eating/wasting disorders and cachexia related to cancer and other conditions, such as cardiovascular disease and chronic obstructive pulmonary disease (Nagaya *et al*. 2004, 2005, Strasser *et al*. 2008, Müller *et al*. 2010, Ali *et al*. 2013, Garcia *et al*. 2015).

Considerable *in vitro* research has investigated the potential role of ghrelin in carcinogenesis and cancer progression, possibly via an autocrine/paracrine pathway (Jeffery *et al*. 2003, Nikolopoulos *et al*. 2010, Chopin *et al*. 2012). One rationale for this research is that endogenous ghrelin stimulates release of GH, which regulates IGF1 concentrations (Jeffery *et al*. 2003, Clemmons 2004). IGF1 has mitogenic and antiapoptotic properties (Khandwala *et al*. 2000), and has been positively correlated in some preclinical, epidemiologic and case–control studies with modestly increased risk of several cancers, particularly hormone-dependent cancers of the breast and prostate (Renehan *et al*. 2004, Pekic & Popovic 2013, Crawley & Holmberg 2014). However, other substantial clinical trial and meta-analysis data have shown no association of IGF1 or its binding proteins (e.g. IGF-binding protein 3 (IGFBP3)) with breast, prostate or colorectal cancers (Renehan *et al*. 2006, Schernhammer *et al*. 2006, Severi *et al*. 2006, Mikami *et al*. 2009, Rowlands *et al*. 2012, Yoon *et al*. 2015), although a positive correlation of insulin/hyperinsulinemia with advanced colorectal cancer has been noted (Yoon *et al*. 2015). Inverse associations of IGFBP3 circulating level with lung cancer (Cao *et al*. 2012), and of IGF1 and placental GH with epithelial ovarian cancer in women aged <55years at diagnosis (Schock *et al.* 2015) have also been observed. Moreover, large, long-term clinical studies of GH therapy have demonstrated no increased risk of neoplasms or recurrent tumors in pediatric patients (Allen *et al.* 1997, Sävendahl *et al.* 2012, Patterson *et al.* 2014, Raman *et al.* 2015) or in adults (Olsson *et al.* 2012, Hartman *et al.* 2013, Brignardello *et al.* 2015, Child *et al.* 2015, Stochholm & Johannsson 2015). Although it has been reported that GH therapy may increase the risk of a second neoplasm in pediatric cancer survivors (Sklar *et al.* 2002), this risk appears to diminish over time (Ergun-Longmire *et al.* 2006).

Regardless of the underlying rationale, numerous *in vitro* studies have investigated the association of ghrelin *per se* with various cancer types, either through or independent of its effect on GH/IGF1 (Jeffery *et al.* 2003, Nikolopoulos *et al.* 2010, Chopin *et al.* 2012). These studies have provided mixed evidence, with most showing increased expression of ghrelin in neoplasms and potential indications of a carcinogenic role, while other studies demonstrated reduced ghrelin expression in tumors and/or a possible antineoplastic effect (Chopin *et al.* 2012). Researchers have cited a need for more *in vivo* studies to clarify whether ghrelin plays a role in cancer (Nikolopoulos *et al.* 2010, Chopin *et al.* 2012). *In vivo* data are essential to illuminate this question since simplified *in vitro* models cannot account for the complex interactions – known and yet to be elucidated – that may lead to clinically important differences in outcomes.

It should be noted that research on plasma ghrelin levels and the effects of ghrelin agonist therapy is complicated by several methodologic factors. Although acyl and des-acyl ghrelin appear to have different actions, most published studies on endogenous ghrelin with regard to cancer have measured total ghrelin, which may be imprecise as to biologic implications (Yoshimoto *et al*. 2002, Akamizu *et al*. 2005, Aydin *et al*. 2008). Indeed, 40–60% of total ghrelin measured using RIA may consist of deacylated C-terminal fragments, possibly as a consequence of the RIA procedure (Akamizu *et al*. 2005). Newer assay methods, such as sandwich-type enzyme immunoassay and other novel adaptations of HPLC, ELISA and RIA can distinguish and separately measure acyl and des-acyl ghrelin (Yoshimoto *et al*. 2002, Hotta *et al*. 2004, Akamizu *et al*. 2005, Aydin *et al*. 2008, Prudom *et al*. 2010). Yet, as noted above, due to the instability of acyl ghrelin, the levels of acyl ghrelin or proportions of acyl and des-acyl ghrelin constituting total ghrelin measures reported in studies may vary substantially. Moreover, ghrelin-receptor agonist therapies are typically designed to bind to the ghrelin receptor in similar manner as acyl ghrelin, thus representing a parallel to acyl but not to des-acyl ghrelin (Garcia *et al*. 2009, Garcia *et al*. 2013). This systematic literature review was conducted to evaluate the current status of the published *in vivo* studies on this topic and to assess the evidence and its implications.

## Methods

**Figure 2:**
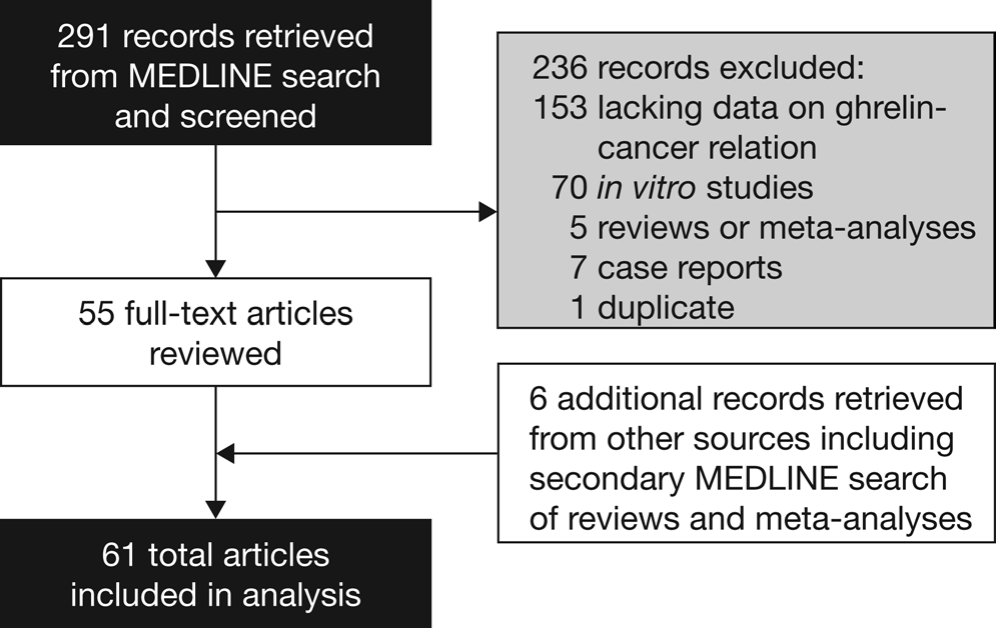
Systematic review flow diagram: *in vivo* research evidence of associations of ghrelin with cancer.

A systematic literature review was conducted to gather and assess the *in vivo* (human and animal) research on the association between endogenous ghrelin levels or exogenously administered ghrelin (including receptor agonists and derivatives), and cancer risk, incidence, growth or metastasis (Fig. 2), following recommended methods for such reviews (Moher *et al.* 2015). We searched National Library of Medicine/MEDLINE PubMed database (http://www.ncbi.nlm.nih.gov/pubmed/; last accessed 9 February 2016) for relevant studies using search terms such as ghrelin, GH, GH secretagogues, and cancer, published during the period of 1 January 1982, through 31 December 2015 (Box 1).

Box 1 **MEDLINE search terms and strings.**

(‘ghrelin’(MeSH Terms) OR ‘ghrelin’(All Fields)) OR ((‘growth hormone’(MeSH Terms) OR (‘growth’(All Fields) AND ‘hormone’(All Fields)) OR ‘growth hormone’(All Fields)) AND (‘growth hormone’(MeSH Terms) OR (‘growth’(All Fields) AND ‘hormone’(All Fields)) OR ‘growth hormone’(All Fields)) AND secretagogues(All Fields)) AND ((‘tumour’(All Fields) OR ‘neoplasms’(MeSH Terms) OR ‘neoplasms’(All Fields) OR ‘tumor’(All Fields)) AND (‘neoplasms’(MeSH Terms) OR ‘neoplasms’(All Fields) OR ‘cancer’(All Fields)) AND (‘neoplasms’(MeSH Terms) OR ‘neoplasms’(All Fields) OR ‘neoplasia’(All Fields))) NOT (‘in vitro techniques’(MeSH Terms) OR (‘vitro’(All Fields) AND ‘techniques’(All Fields)) OR ‘in vitro techniques’(All Fields) OR ‘vitro’(All Fields) OR ‘in vitro’(All Fields)) NOT (‘review’(Publication Type) OR ‘review literature as topic’(MeSH Terms) OR ‘review’(All Fields)) AND ((‘1982/01/01’(PDAT) : ‘2015/12/31’(PDAT)) AND (‘humans’(MeSH Terms) OR ‘animals’(MeSH Terms:noexp)) AND English(lang))

Eligible items were published, original research, peer-reviewed, *in vivo* studies (including letters) that reported an association between endogenous ghrelin levels, the administration of ghrelin or ghrelin-receptor agonists, or ghrelin gene polymorphisms, with cancer incidence, presence, growth or metastasis, excluding noncancerous growths (e.g. polycystic ovary syndrome). Noncancer controls or a reference range of physiologic ghrelin levels were required for association with incidence or presence of cancer, but not for tumor growth or metastasis. Other eligibility requirements included an accessible abstract for review and publication in English. If repeat studies were performed in the same study population, only the later study was included. Case reports/series were excluded so as to maintain minimum standards of study design/size. Review articles and meta-analyses were also excluded so as to allow for direct evaluation of original study data.

In addition, reference lists of the articles selected for analysis were reviewed for additional original research citations (i.e. ancestry search). A secondary search was also conducted using the same search terms and limits as described above (Box 1) to identify reviews and meta-analyses only (excluded from the initial search), with review of the reference lists of articles thus obtained for original research citations not previously identified. Results were described narratively, without meta-analysis of the data.

## Results

The initial search retrieved 291 records, of which 236 articles were excluded because they fell in the following categories: case reports (7); lack of data on ghrelin/ghrelin-receptor agonists and cancer (153); *in vitro* studies (70); reviews or meta-analyses (5); and duplicate articles (1) (Fig. 2). A total of 55 articles from the initial search were judged eligible for inclusion in addition to 6 articles identified from other sources (e.g. reference lists of original research articles, reviews and meta-analyses). Thus, a total of 61 original research articles were included in the analysis (Fig. 2).

Of these 61 studies, 50 examined endogenous levels and actions of ghrelin or polymorphisms of ghrelin genes and 11 reported the effects of exogenously administered ghrelin or ghrelin-receptor agonist therapy in association with cancer. A wide range of cancer types/locations were studied, including lung, prostate, breast, leukemic, head and neck, reproductive and neuroendocrine, although most commonly those of the gastrointestinal system (Tables 1 and 2). Ten studies investigated the association between ghrelin gene polymorphisms and cancer risk (Table 1).

**Table 1:** *In vivo* studies of endogenous ghrelin in cancer.

**Cancer type**	**Species**						
		**Human** (*n*)					
Citation	**Animal**	**Patients**	**Controls**	**Ghrelin measure**	**Plasma/serum level (↑/↓) vs controls/effect on risk** (inverse/positive)	**Effect of genetic variant(s) on cancer risk**	**Association of ↑ plasma ghrelin with tumor stage/growth/metastasis**
**Acute lymphoblastic leukemia**							
Moschovi *et al*. (2008)^a^		9	9	Acyl	Decreased*	NR	Inverse*
**Breast**							
Dossus *et al*. (2008)^a^		1359	2389	NR	NR	No association/positive*	NR
Feigelson *et al*. (2008)^a^		648	659	NR	NR	No association	NR
Wagner *et al*. (2006)		798	1011	NR	NR	No association/inverse*	NR
**Cancer (various)**							
Garcia *et al*. (2006)		31	25	Acyl	Increased*^b^	NR	NR
Laurila *et al*. (2014)^a,c^		491	513	Total	No association	No association/inverse*^d^	NR
Legakis *et al*. (2009)		30	27	Total	Decreased*	NR	NR
Mondello *et al*. (2014)		140	30	Total	Increased*	NR	NR
Till *et al*. (2015)	Rats			Total/Acyl	Increased*^e^	NR	NR
**Colorectal**							
Campa *et al*. (2010)		197	217	NR	NR	No association/inverse*	NR
D’Onghia *et al*. (2007)^a^		29	50	Total	Decreased*	NR	Inverse*
Kemik *et al*. (2010)		126	36	Total	Decreased*	NR	NR
Mahmoudi *et al*. (2014)		1249	1319	NR	NR	No association	NR
Nikolopoulos *et al*. (2014)^a^		95	39	Total	Increased*	NR	NR
**CP and pituitary**							
Holmer *et al*. (2010)		42	42	Total	Decreased*	NR	Inverse*
Roemmler-Zehrer *et al*. (2014)		40	40^f^	Total	Decreased*	NR	NR
Roth *et al*. (2011)^a^		27	27	Total	Decreased*	NR	No association
Trivin *et al*. (2009)		27^g^	0	Total	NR	NR	Inverse*
**Endometrial**							
Fung *et al*. (2013)	Mice			Total	NR	NR	Positive*
**Esophageal**							
De Martel *et al*. (2007)^a^		52	156	Total	Inverse^h^	NR	NR
Doecke *et al*. (2008)^a^		774	1352	NR	NR	No association	NR
Miyazaki *et al*. (2012)^a^		25	0	Total	NR	NR	No association
Murphy *et al*. (2012)		82	82	Total	Inverse*	NR	NR
**Gastric/gastrointestinal**							
Benedix *et al*. (2011)^a^		17	86	Total	No association	NR	NR
Huang *et al*. (2007)a		78	24	Total	No association	NR	NR
Isomoto *et al*. (2005)^a^		23	249^i^	Total	No association	NR	Positive*
Kemik *et al*. (2012)		148	40	Total	Decreased*	NR	NR
Murphy *et al*. (2011)^a^		359	441	Total	Inverse*	NR	NR
Sadjadi *et al*. (2013)		220	220	Total	Inverse*^j^	NR	NR
Tsolakis *et al*. (2008)		20	RR	Total	No association	NR	NR
Zub-Pokrowiecka *et al*. (2010)		25	10	Total	No association	NR	NR
Zub-Pokrowiecka *et al*. (2011)		45	25	Total	Decreased*	NR	NR
**Head and neck**							
Ozsoy *et al*. (2015)		40	20	NR	No association	NR	NR
**Liver**							
Ataseven *et al*. (2006)		22	25	Total	Increased*	NR	NR
Lin and Yin (2007)		40	20	Total	Decreased*	NR	Inverse*
Motawi *et al*. (2013)		79	40	NR	NR	Positive*	NR
Pourtau *et al*. (2011)^a^	Rats			Total	Decreased	NR	NR
**Lung**							
Karapanagiotou *et al*. (2009)^a^		101	60	Total	Increased*	NR	No association
Kerenidi *et al*. (2013)^a^		80	40	Total	Increased*	NR	No association
Shimizu *et al*. (2003)^a^		43	21	Total	No association	NR	NR
Tsao *et al*. (2007)		50	16	Total	Increased*	NR	Positive*
**Ovarian**							
Markowska *et al*. (2009)		53	32	Acyl/Total	No association/increased*^k^	NR	NR
**Neuroendocrine**							
Wang *et al*. (2007)		35	RR	Total	Increased*	NR	Positive*
**Non-Hodgkin lymphoma**							
Skibola *et al*. (2005)^a^		458	812	NR	NR	No association/Inverse*	NR
**Pancreatic**							
Corbetta *et al*. (2003)		40^l^	35	Total	No association	NR	No association
Ekeblad *et al*. (2007)		26	5	Total	No association	NR	NR
Zhang *et al*. (2014)		173	476	NR	NR	No association	NR
**Prostate**							
Mungan *et al*. (2008)^a^		30	50^m^	Total	No association	NR	No association
Malendowicz *et al*. (2009)^a^		18	16^m^	Acyl/Des-Acyl/Total	No association/Increased*^k^	NR	NR
**Thyroid**							
Morpurgo *et al*. (2005)^a^		22	15	Total	No association	NR	No association

The significance of increased, decreased or unchanged serum/plasma ghrelin levels in cancer with regard to incidence, progression or prognosis remains unclear. *P≤0.05. CP, craniopharyngioma; NR, not reported; RR, reference range.

a Adjusted for body mass index/obesity/cachexia/weight loss

b Ghrelin levels were also significantly correlated with weight loss; relationship of plasma ghrelin with cancer independent of weight loss was not reported

c Prospective, 19-year, population-based study in 491 hypertensive and 513 nonhypertensive, healthy subjects (no cancer patients at baseline)

d Significantly decreased risk in healthy subjects only (no association for hypertensive subjects)

e Significant increase for human neuroblastoma, nonsignificant increase for human hepatoblastoma

f Control patients had nonfunctioning pituitary adenoma

g Study compared 27 patients with grade 0 (*n*=7), grade 1 (*n*=8), or grade 2 (*n*=12) craniopharyngioma

h Inverse association seen in overweight subjects only

i Controls included patients/subjects with acute gastritis, benign gastric polyp, chronic gastritis, duodenal ulcer, gastric ulcer, or normal gastric mucosa

j Inverse relationship statistically significant for serum ghrelin and gastric noncardia cancer, gastric cardia cancer, and esophageal squamous cell carcinoma but not for gastric adenocarcinoma

k Acyl-ghrelin concentration was significantly increased and total ghrelin level not different in cancer patients versus controls

l Sixteen patients had gastrointestinal carcinoid and 24 had pancreatic tumor

m Controls had benign prostate hyperplasia.

**Table 2:** *In vivo* studies of exogenous ghrelin/ghrelin-receptor agonist treatment in cancer.

		**Species**			
			**Human** (*n*)		
**Citation**	**Condition treated/outcome assessed** (cancer type/model)	**Animal model**	**Treatment**	**Control**	**Effect on tumor incidence or growth**
Chen *et al.* (2015)^a^	Cancer- and cisplatin-induced muscle wasting (Lewis lung carcinoma)	Mice			No effect
DeBoer *et al.* (2007)^a^	Cancer cachexia (MC sarcoma)	Rats			No effect
Garcia *et al.* (2015)^a^	Cancer cachexia (various, advanced)		44	38	No effect
Hanada *et al.* (2003)^a^	Cancer cachexia (human melanoma)	Mice			No effect
Hiura *et al.* (2012)^a^	Chemotherapy-induced appetite/eating disorders (esophageal)		21	21	No effect
Kawaguchi *et al.* (2015)^a^	Tumorigenesis (intestinal)	Mice			No effect/inverse*
Lundholm *et al.* (2010)	Cancer weight loss (gastrointestinal)		17^b^	14^c^	No effect
Northrup *et al.* (2013)^a,d^	Tumor growth (lung)	Mice			No effect
Strasser *et al.* (2008)^a^	Cancer anorexia/cachexia (various, advanced)		11^e^	9^f^	No effect
Tsubouchi *et al.* (2014)^a^	Cancer cachexia (lung)	Mice			No effect
Wang (2006)^a^	Cancer cachexia (MC sarcoma)	Mice			No effect

The significance of increased, decreased or unchanged serum/plasma ghrelin levels in cancer with regard to incidence, progression or prognosis remains unclear.

* *P*<0.05 in the murine azoxymethane/dextran sodium sulfate-induced inflammation-associated colon carcinogenesis model. MC, methylcholanthrene.

a Placebo (e.g. saline) was administered to at least one control group or used in crossover design

b High-dose ghrelin (13±1μg/kg daily)

c Low-dose ghrelin (0.7±0.4μg/kg daily)

d Active treatment groups received either ghrelin 2mg/kg intraperitoneally, or anamorelin 3, 10 or 30mg/kg orally

e High-dose ghrelin (8μg/kg daily) on days 1 and 8 and placebo on days 4 and 11 or vice versa

f Low-dose ghrelin (2μg/kg daily) on days 1 and 8 and placebo on days 4 and 11 or vice versa.

An overall count showed that 46 (75.4%) of the studies, including all 11 involving exogenous ghrelin/ghrelin-receptor agonist treatment, reported either a null (no statistically significant difference) or inverse association of ghrelin or ghrelin genetic variants with cancer risk, presence or growth; 9 (14.8%) studies reported positive associations; and 6 (9.8%), including 4 gene studies, reported both negative or null and positive associations (Tables 1 and 2).

### Endogenous ghrelin noninterventional studies

Of the 49 noninterventional studies, 46 were clinical and 3 in animal models, including the 10 studies of genetic polymorphisms of ghrelin genes and cancer, of which 7 reported null or inverse results while 3 showed a link to increased risk (Table 1). However, the significance and physiologic function of differences in the serum or plasma ghrelin levels of cancer patients vs controls remained unclear, with study authors suggesting various hypotheses, most commonly based on the known metabolic actions of ghrelin. Although the studies of ghrelin levels and cancer risk generally used healthy controls, few made reference to physiologic ranges of plasma/serum ghrelin. All but three studies reporting serum/plasma ghrelin levels reported total ghrelin. Two studies reported both total ghrelin and acyl ghrelin, both having contrasting results for each measure in cancer patients vs controls (Malendowicz *et al*. 2009, Markowska *et al*. 2009), and one study reported results for acyl ghrelin only (Garcia *et al*. 2006).

In the largest, gastrointestinal cancer group (including gastric, esophageal and colorectal cancers), several population-based, long-term, prospective studies showed an inverse association of baseline ghrelin level with risk of gastric, esophagogastric and esophageal cancer incidence (de Martel *et al.* 2007, Murphy *et al.* 2011, Murphy *et al.* 2012). These included two nested case–control studies that used logistic regression analysis and multivariate adjustment within the Finnish Alpha-Tocopherol, Beta-Carotene (ATBC) Cancer Prevention study, a randomized, placebo-controlled, primary prevention study in 29,133 Finnish male smokers. One study, in 261 patients with gastric noncardia adenocarcinoma (GNCA) and 98 with esophagogastric junctional adenocarcinoma (EGJA) vs 441 controls found significant inverse correlations of serum ghrelin level with incidence of both cancers (GNCA adjusted odds ratio (OR) 1.75, 95% CI: 1.49–2.04; EGJA adjusted OR: 1.56, 95% CI: 1.29–1.89; *P*<0.001 for both) (Murphy *et al.* 2011). The other ATBC study, in 82 patients with esophageal squamous cell carcinoma (ESCC) vs 82 controls matched for age and date of blood draw, reported a multivariate OR of 6.83 (95% CI: 1.46–31.84) for ESCC in individuals in the lowest quartile of baseline serum ghrelin vs those in the highest quartile (*P*=0.005 for trend). The results for both analyses remained significant for cancers occurring more than 10years after baseline ghrelin measurement (Murphy *et al.* 2011, 2012).

Another nested case–control study within a population of 128,992 enrolled in a public health program between 1964 and 1969, including 52 cases of esophageal cancer identified by the year 2000, found a nonsignificant correlation of high serum ghrelin with reduced risk of esophageal cancer in overweight subjects vs controls matched for age, race, sex and date/site of blood draw (*P*=0.09 for trend), adjusted for BMI and *Helicobacter pylori* infection (De Martel *et al.* 2007). In addition, an Australian case–control genetic study (774 esophageal cancer cases vs 1352 controls) found no correlation of the obesity-related ghrelin SNPs sampled (rs468677 (L90Q), rs696217 (M72L)) with esophageal cancer incidence (Doecke *et al.* 2008).

Several studies in gastric, gastroesophageal, and colorectal cancers found either no difference in serum ghrelin levels in cancer patients vs controls (Isomoto *et al.* 2005, Huang *et al.* 2007, Tsolakis *et al.* 2008, Benedix *et al.* 2011, Zub-Pokrowiecka *et al.* 2011), or lower ghrelin levels in cancer patients (Kemik *et al.* 2012, Sadjadi *et al.* 2013) (Table 1). However, in one of these studies, ghrelin levels were significantly higher in patients with undifferentiated adenocarcinomas (*n*=9) than in patients with differentiated tumors (*n*=14) (*P*<0.005) (Isomoto *et al.* 2005).

Two of three studies investigating serum ghrelin levels in colon or colorectal cancer found significantly decreased levels in the cancer patients vs controls (D’Onghia *et al.* 2007, Kemik *et al.* 2010), including one, in 29 patients with colorectal cancer and 50 controls, that also found ghrelin serum levels were significantly inversely associated with tumor stage (D’Onghia *et al.* 2007) (Table 1). However, another study reported significantly higher total serum ghrelin levels in 95 patients with colon cancer vs those in 39 healthy controls matched for age, gender and BMI; serum ghrelin level was also positively correlated with tumor size and end-stage vs initial stage tumors, and inversely associated with tumor differentiation, but not correlated with patient survival, independent of Dukes stages (Nikolopoulos *et al.* 2014). Acknowledging the many physiologic and hormonal factors regulating ghrelin serum levels, the authors concluded that it remained unclear whether ghrelin promoted or inhibited carcinogenesis (Nikolopoulos *et al.* 2014).

Of two clinical studies in patients with liver cancer, one Turkish study reported significantly increased serum ghrelin levels in 22 patients with hepatocellular carcinoma (HCC) due to hepatitis B or D virus and similarly increased levels in 23 patients with cirrhosis vs 25 control subjects (Ataseven *et al.* 2006) (Table 1). Since 19 of the 22 HCC patients also had cirrhosis, and had ghrelin levels similar to the cirrhosis cohort, the authors interpreted the increased ghrelin as a response to cirrhosis-related catabolic conditions (Ataseven *et al.* 2006). In contrast, the other study reported a significantly reduced ghrelin concentration in 40 Taiwanese patients with HCC vs 20 healthy controls, and an inverse correlation of ghrelin levels with HCC stage (Lin & Yin 2007).

Among four clinical studies of endogenous ghrelin in heterogeneous populations of patients with various cancers (including gastric, pancreatic, lung, breast, multiple myeloma, lymphomas, head and neck, rectal, adenocarcinoma and gynecological), one reported increased total ghrelin (Mondello *et al*. 2014) and one increased acyl ghrelin level (Garcia *et al*. 2006) in the cancer patients vs controls. The authors of both studies attributed these results largely to a physiologic ghrelin response to weight loss or cachexia, which was present in most patients (Garcia *et al.* 2006, Mondello *et al.* 2014) (Table 1). A large, 19-year, population-based prospective follow-up study in 491 hypertensive and 513 control subjects (cardiovascular disease incidence was a parallel outcome of the study) found that baseline plasma ghrelin level had no association with cancer deaths or hospital events in either cohort (Laurila *et al.* 2014). In addition, a study in 30 patients with various advanced, inoperable cancers, primarily gastric and pancreatic, and weight loss with malnutrition, found that plasma ghrelin was significantly lower (*P*<0.001) in the cancer patients vs 27 healthy subjects (Legakis *et al.* 2009). The decreased ghrelin in this study was attributed to the severity and progression of cancer with possible involvement of multiorgan failure, particularly since no correlation between ghrelin level and histological type of malignancy was observed (Legakis *et al.* 2009).

Of the four clinical studies in lung cancer, three reported significantly increased serum ghrelin levels vs controls in cancer patients (Tsao *et al.* 2007, Karapanagiotou *et al.* 2009, Kerenidi *et al.* 2013) (Table 1). However, one study found that while serum ghrelin was not different overall between lung cancer patients (*n*=43; 21 with cachexia and 22 without cachexia) vs controls (*n*=21), they were significantly increased in the patients with cachexia vs those without cachexia (Shimizu *et al.* 2003) (Table 1). The study authors suggested that the ghrelin increase was a compensatory mechanism triggered by cachectic catabolic–anabolic imbalance (Shimizu *et al.* 2003). Of the other studies, one found that serum ghrelin levels were increased in lung cancer patients (*n*=80) vs healthy controls (*n*=40), although only 17 of the patients had weight loss, and the study groups were matched for BMI (Kerenidi *et al.* 2013). However, serum ghrelin level had no association with survival, while leptin was independently correlated with significantly shorter survival. Noting the mutually antagonistic metabolic actions and other effects of leptin and ghrelin, the authors suggested the elevated ghrelin could be a protective mechanism to neutralize leptin and thus impede cancer progression (Kerenidi *et al.* 2013). Another of the studies reporting increased serum ghrelin levels, in 101 patients with lung cancer vs 60 healthy controls, found this increase was independent of BMI, although patients with weight loss had significantly higher ghrelin level than those without weight loss (Karapanagiotou *et al.* 2009). Serum ghrelin level also had no association with survival. The authors postulated that the serum ghrelin increase occurred as a compensatory response to weight loss in cachectic patients, and as an anti-inflammatory response to a lung cancer-induced ‘systemic inflammation cascade’ (Karapanagiotou *et al.* 2009). Authors of the third study reporting increased ghrelin, in 40 Taiwanese patients with lung cancer vs 16 controls, postulated that the ghrelin increase was a compensatory mechanism to increase energy/nutrition, particularly B_2_ and B_6_ vitamin levels, which were greatly reduced (Tsao *et al.* 2007).

Four studies investigated ghrelin levels in patients with craniopharyngioma (CP) – which is associated with obesity, metabolic syndrome and GH deficiency – and pituitary cancer (Trivin *et al.* 2009, Holmer *et al.* 2010, Roth *et al.* 2011, Roemmler-Zehrer *et al.* 2014) (Table 1). Three of the studies reported significantly reduced ghrelin levels in CP patients vs controls (Holmer *et al.* 2010, Roth *et al.* 2011, Roemmler-Zehrer *et al.* 2014), and two reported a significant inverse association of serum ghrelin level with CP tumor growth (Trivin *et al.* 2009, Holmer *et al.* 2010). While two of these studies were controlled by age and gender but not BMI (Holmer *et al.* 2010, Roemmler-Zehrer *et al.* 2014), the one study that did match CP patients (*n*=27) and controls (*n*=27) for BMI as well as age and gender found that obese CP patients had lower ghrelin levels than obese controls (Roth *et al.* 2011).

Among two studies of ghrelin levels in patients with pancreatic cancer, both found no difference in the plasma ghrelin levels of the cancer patients vs controls (Corbetta *et al.* 2003, Ekeblad *et al.* 2007), including one that also found no correlation of ghrelin level with cancer progression (Corbetta *et al.* 2003) (Table 1). Three studies assessed associations of multiple ghrelin gene polymorphisms with breast cancer risk (Wagner *et al.* 2006, Dossus *et al.* 2008, Feigelson *et al.* 2008). A European study in 1359 breast cancer cases and 2389 matched controls found that carriers of the ghrelin rs171407-G allele had a significantly increased breast cancer risk (OR: 1.2, 95% CI: 1.0–1.4; *P*=0.02) (Dossus *et al.* 2008). A Polish and German study of various hormonal gene SNPs with a proven or potential functional effect, in 798 breast cancer cases and 1011 controls, found a decreased risk of cancer associated with two rare ghrelin haplotypes, GGAC (OR: 0.05, 95% CI: 0.01–0.79; *P*=0.001) and GGAT (OR: 0.23, 95% CI: 0.04–1.13; *P*=0.04) (Wagner *et al.* 2006). The third study evaluated tagging SNPs of obesity-related genes, in 648 breast cancer cases and 659 controls from the American Cancer Society Cancer Prevention Study II Nutrition Cohort, and found no association between any ghrelin gene SNPs and breast cancer (Feigelson *et al.* 2008).

Of two studies in prostate cancer, 1 in 30 patients vs 50 controls with benign prostate hyperplasia (BPH) found no association of ghrelin level with presence or progression of cancer (Mungan *et al.* 2008) (Table 1). The other study found that total plasma ghrelin concentrations were similar, but acyl ghrelin levels and ratios of acyl ghrelin to total ghrelin and to obestatin were significantly higher, in 18 patients with prostate cancer vs 12 controls with BPH (Malendowicz *et al.* 2009). A study in 53 patients with ovarian cancer reported similar findings of significantly elevated acyl ghrelin and acyl to total ghrelin ratio, but no difference in total ghrelin plasma levels, in the cancer patients vs 32 controls (Markowska *et al.* 2009). However, the authors of this study stated that the lack of evidence of a human ovarian ghrelin receptor made it doubtful that ghrelin was directly linked to ovarian carcinogenesis (Markowska *et al.* 2009).

Single studies also reported no association of serum ghrelin levels with head and neck cancers (Ozsoy *et al.* 2015) and thyroid cancer (Morpurgo *et al.* 2005), and inverse correlations of ghrelin with both presence of acute lymphoblastic leukemia and tumor burden (Moschovi *et al.* 2008) (Table 1). A study in 35 patients with neuroendocrine tumors found that serum ghrelin level was significantly elevated as compared with a physiologic reference range in patients with hepatic metastases, which was interpreted as a co-release of ghrelin from neuroendocrine tumors generated as a physiological mechanism to maintain appetite and BMI (Wang *et al.* 2007).

### Exogenous ghrelin interventional studies

Of the 11 studies of exogenous ghrelin or ghrelin-receptor agonist intervention (acyl ghrelin therapies) over periods ranging from 1 to 12 weeks in patients or animals with cancer in this sample, 10 found no effect of the therapy on tumor growth or markers vs placebo or between different dose groups (Hanada *et al.* 2003, Wang *et al.* 2006, DeBoer *et al.* 2007, Strasser *et al.* 2008, Lundholm *et al.* 2010, Hiura *et al.* 2012, Northrup *et al.* 2013, Tsubouchi *et al.* 2014, Chen *et al.* 2015); one reported both no effect and an inverse correlation of ghrelin in different animal models of cancer (Kawaguchi *et al.* 2015) (Table 2).

Among the four clinical trials, one compared ghrelin therapy with placebo for treatment of chemotherapy-induced eating disorders in 21 patients with esophageal cancer vs 21 controls (Hiura *et al.* 2012); another assessed effects of high-dose ghrelin (*n*=17; 13±1μg/kg daily) vs low-dose ghrelin (*n*=14; 0.7±0.4μg/kg daily) for weight loss in patients with gastrointestinal cancers (Lundholm *et al.* 2010); and a third compared the effects of ghrelin at high dose (*n*=11; 8μg/kg daily) and low dose (*n*=9; 2μg/kg daily) or placebo alternately in a crossover design for treatment of anorexia/cachexia related to various cancer (Strasser *et al.* 2008). The largest of these studies (*n*=82) was a pooled analysis of two similarly designed Phase II, randomized, double-blind, placebo-controlled, multicenter trials of treatment with anamorelin, a ghrelin-receptor agonist, for cancer cachexia in patients with various advanced, incurable cancers (breast, colon, lung, genitourinary and others; Eastern Cooperative Oncology Group Score of ≤2) and cachexia defined as a weight loss of ≥5 within the previous 6months (Garcia *et al.* 2015). The incidence of neoplasms or tumor progression (benign, malignant or unspecified) was similar over this 12-week trial in both the anamorelin (*n*=44) and placebo (*n*=38) groups.

Two of the seven animal studies assessed the effect of ghrelin on tumor growth as the primary outcome (Northrup *et al.* 2013, Kawaguchi *et al.* 2015). In one study, ghrelin administration had a significant inverse effect on tumor growth in a murine model of inflammation-associated colon carcinogenesis (*P*<0.0001), although it had no effect in a genetic susceptibility model (Kawaguchi *et al.* 2015). Deletion of the ghrelin gene had no significant effect on tumorigenesis in either model. In the other study, nude mice with established, implanted A549 nonsmall cell lung cancer tumors were administered either saline, or ghrelin 2mg/kg or anamorelin dosed at 3mg/kg orally (po), 10 or 30mg/kg po (Northrup *et al.* 2013). While tumor growth progressed steadily over the 28-day trial period, no differences in this parameter were observed between the treatment groups, despite increases in GH and IGF1 after ghrelin and anamorelin treatment (Northrup *et al.* 2013).

## Discussion

### Comparison with *in vitro* findings

This systematic analysis of *in vivo* studies of associations of ghrelin with cancer provides evidence that is approximately the reverse of that suggested by published *in vitro* studies. Whereas the majority of *in vitro* studies suggest upregulation of ghrelin in cancer tissues, the majority (over 70%) of *in vivo* studies have shown null or inverse relations of ghrelin to cancer (Tables 1 and 2). Indeed, two clinical studies in this review that assessed both *in vitro* and *in vivo* levels of ghrelin in patients with cancer reported that despite findings of high ghrelin expression in tumor tissue, plasma ghrelin measures were either similar to those of healthy controls (Ekeblad *et al.* 2007) or within the reference range for this measure (Tsolakis *et al.* 2008). On the other hand, an animal study did report similar *in vitro* and *in vivo* findings that experimental silencing or ‘knockdown’ of the ghrelin-receptor expression in murine models of endometrial cancer led to reduced tumor growth (Fung *et al.* 2013).

### The ghrelin/GH/IGF1 axis

The *in vivo* data in this review provided little support for the hypothesis, noted above, that ghrelin could promote carcinogenesis via the GH/IGF1 pathway in an autocrine/paracrine manner (Jeffery *et al.* 2003). The absence of carcinogenic effects demonstrated in any of the clinical or animal trials of exogenous ghrelin or ghrelin-receptor agonist therapy is also consistent with the considerable clinical data showing no association of GH therapy with increased risk of cancer in children (Allen *et al.* 1997, Sävendahl *et al.* 2012, Patterson *et al.* 2014, Raman *et al.* 2015) or in adults (Olsson *et al.* 2012, Hartman *et al.* 2013, Brignardello *et al.* 2015, Child *et al.* 2015, Stochholm & Johannsson 2015). Of the four clinical trials of exogenous ghrelin/ghrelin-receptor agonist therapy, three reported no significant differences in GH/IGF1 levels vs placebo (Strasser *et al.* 2008, Lundholm *et al.* 2010, Hiura *et al.* 2012). The 12-week trial of anamorelin reported significant increases in IGF1 and IGFBP3 levels vs placebo (*P*≤0.0002), although these concentrations remained within the normal ranges (Garcia *et al.* 2015). A nonsignificant increase in IGF1 level was also observed with anamorelin vs placebo in the murine lung cancer study (Northrup *et al.* 2013). General long-term clinical safety and efficacy data for anamorelin have recently become available. Data from ROMANA 3 (*n*=513), a 12-week safety extension study of two randomized, placebo-controlled, 12-week, Phase III trials of anamorelin, in patients with unresectable stage III or IV nonsmall cell lung cancer with cachexia given anamorelin (*n*=345 (67.3%)) or placebo (*n*=168 (32.7%)), totaling 24weeks of exposure, also showed no differences in treatment-emergent adverse events, including deaths, between the treatment and placebo groups; none of the deaths in the study were judged treatment related (Currow *et al.* 2015). In addition, subsequent to the initial systematic search period (1 January 1982, through 31 December 2015), longer-term survival data have been published for anamorelin, which showed no difference in median survival over 1year (8.90months, 95% CI: 8.3–9.8) compared with placebo (9.17months, 95% CI: 7.9–11.0; hazard ratio 1.06, 95% CI: 0.89–1.26; *P*=0.47) (Temel *et al*. 2016).

### Variations and discrepancies in serum ghrelin levels

Although the *in vivo* studies included in this analysis provided little evidence of a carcinogenic role of ghrelin, many indicated changes in serum ghrelin levels in the cancer environment, which are yet to be elucidated. Increased serum ghrelin in cancer was most frequently attributed to known, noncarcinogenic, physiologic actions of ghrelin adapted to a cancer environment. These included a compensatory response to cancer weight loss and cachexia (Shimizu *et al.* 2003, Garcia *et al.* 2006, Wang *et al.* 2007, Karapanagiotou *et al.* 2009, Mondello *et al.* 2014), which has been previously reported in the literature (Wolfe *et al.* 2006); a mechanism to improve nutritional status (Tsao *et al.* 2007); an anti-inflammatory response to concomitant conditions such as cirrhosis (Ataseven *et al.* 2006); a protective response to cancer-induced inflammation (Karapanagiotou *et al.* 2009); or a mechanism to neutralize potential carcinogenic actions of leptin (Kerenidi *et al.* 2013). Additional cancer-related factors that may influence ghrelin levels, reported in the broader literature, include chemotherapy-induced inflammation and cancer-associated dyspepsia (Malik *et al.* 2008), cancer-associated inflammation (Guney *et al.* 2007, Kawaguchi *et al.* 2015) and postoperative, acute-phase stress (Maruna *et al.* 2008).

Other studies reported inverse correlations of serum ghrelin levels and risk of cancer (De Martel *et al.* 2007, Murphy *et al.* 2011, 2012, Sadjadi *et al.* 2013), as well as decreased serum ghrelin levels in cancer patients vs controls (D’Onghia *et al.* 2007, Lin & Yin 2007, Moschovi *et al.* 2008, Legakis *et al.* 2009, Kemik *et al.* 2010, 2012, Roth *et al.* 2011, Zub-Pokrowiecka *et al.* 2011) (Table 1). The reasons for decreased ghrelin levels in cancer in this analysis were also unclear, but are hypothesized to involve cancer-associated impairment of normal physiologic regulation of ghrelin production and response to other factors (Shimizu *et al.* 2003, Huang *et al.* 2007, Legakis *et al.* 2009, Zub-Pokrowiecka *et al.* 2011). Studies suggest that physiologic ghrelin responses to fasting and postprandial states are blunted or nonexistent in cancer patients (Roth *et al.* 2011, Zub-Pokrowiecka *et al.* 2011) and in rats with hepatoma cells (Portau *et al.* 2011). In addition, cancer-related surgeries such as gastrectomy and esophagectomy are associated with decreased serum ghrelin levels, relative to presurgery levels (Zub-Pokrowiecka *et al.* 2011, Miyazaki *et al.* 2012).

Several studies in this analysis reported inverse associations of ghrelin to tumor progression, suggesting a protective effect (D’Onghia *et al.* 2007, Lin & Yin 2007, Moschovi *et al.* 2008, Trivin *et al.* 2009, Holmer *et al.* 2010), which could involve anti-inflammatory actions (Bataar *et al.* 2011, Kawaguchi *et al.* 2015). In preclinical models, ghrelin has demonstrated significant anti-inflammatory actions, including inhibition of the production of proinflammatory cytokines (Dixit *et al.* 2004, Li *et al.* 2004, Gonzalez-Rey *et al.* 2006, Chen *et al.* 2015).

### Genetic findings

This analysis found no clear, net effect of ghrelin gene polymorphisms on cancer risk (Table 1), which is consistent with the conclusions of previous meta-analyses that evaluated the same genetic study data across ghrelin and ghrelin-receptor SNPs in patients with varied cancer types (Pabalan *et al.* 2014, Zhu *et al.* 2015). Follow-up analyses of results showing either positive (Dossus *et al.* 2008, Motawi *et al.* 2013) or inverse (Skibola *et al.* 2005, Wagner *et al.* 2006, Laurila *et al.* 2014) associations of ghrelin gene SNPs with cancer risk warrant further investigation (Table 1).

### Limitations of this analysis

Although 61 studies were included in this analysis, the greatest number investigated gastrointestinal system cancers and additional studies in other cancers are needed to obtain a more complete picture of the potentially complex actions of ghrelin. Since most of the clinical studies were conducted in white populations in North America or Europe, studies with greater ethnic and racial diversity, larger sample sizes, and prospective designs are also needed. The studies were also inconsistent in use of multivariable analysis and adjustment, particularly involving factors such as BMI and cachexia, to isolate the actions of ghrelin on incidence and growth of cancer, and in use of referent physiologic ghrelin levels for confirmation/clarification of findings. The questions also remain as to whether serum/plasma total or acyl ghrelin is the most relevant measure with reference to cancer (i.e. association with cancer risk, presence, or progression). Finally, long-term prospective studies of exogenous ghrelin or ghrelin-receptor agonist administration that focus on tumor progression as a primary outcome are needed.

## Conclusions

The available *in vivo* study evidence suggests that ghrelin has either a null or inverse association with risk or progression of most cancers, although there is not enough evidence to confirm that this holds for all cancers. These findings also suggest that the safety profile of ghrelin or ghrelin-receptor agonist therapy may be favorable for treatment of cachexia and wasting in patients with cancer. Additional large-scale prospective clinical trials are warranted to further elucidate the effects of ghrelin on tumors and general activity in various cancer states, and to evaluate the safety and benefits of ghrelin/ghrelin-receptor agonist treatment in patients with cancer.

## Declaration of interest

The content of this manuscript and the decision to publish are solely the responsibility of the authors and does not necessarily represent the official views of the Department of Veterans Affairs or the National Institutes of Health. S Sever has declared no conflicts of interest.

## Funding

D L White receives research support from the National Institute of Diabetes Digestive and Kidney Diseases (R03 DK095082, PI: White) and the Michael E DeBakey Veterans Affairs Health Services Research Center of Innovations (CIN13-413). J M Garcia has received consulting or advisory fees from Aeterna Zentaris and Helsinn Therapeutics (USA), and research grants from the Department of Veterans Affairs (MERIT grants bib1-BX000507 and bib1 CX000174, and the NIA T32AG000183 and AG040583), Aeterna Zentaris, and Helsinn Therapeutics (USA). Support for developing this manuscript was provided by Helsinn Therapeutics.
